# Editorial: The Role of Sex in Heart Failure and Transplantation

**DOI:** 10.3389/fcvm.2021.690438

**Published:** 2021-05-25

**Authors:** Manuel Martínez-Sellés, Ana Ayesta, Beatriz Díaz-Molina, Antoni Bayes-Genis, Adrian Baranchuk

**Affiliations:** ^1^Servicio de Cardiología, Hospital General Universitario Gregorio Marañón, CIBERCV. Universidad Europea, Universidad Complutense, Madrid, Spain; ^2^Servicio de Cardiología, Hospital Universitario Central de Asturias, Oviedo, Spain; ^3^Servicio de Cardiología, Hospital Universitari Germans Trias i Pujol, CIBERCV, Universidad Autónoma de Barcelona, Badalona, Spain; ^4^Kingston Health Science Center, Division of Cardiology, Queen's University, Kingston, ON, Canada

**Keywords:** heart failure, heart transplantation, sex, prognosis, gender

The epidemic of heart failure (HF) is increasing, mainly due to population aging. As women tend to develop HF at an older age compared to men (1), the prevalence of HF will probably grow at a higher speed in females than in males. Sex-related differences in HF have been described and include epidemiology, etiology, diagnosis, treatment, and prognosis. Unfortunately, women are underrepresented in HF trials and the source of most of these differences is unclear. Women have a better age-adjusted prognosis but survival gains were less in women over the last two decades (2). In addition, women experience worse quality of life during and after HF hospitalization (3). This Research Topic aims to focus on sex-related factors in HF and transplantation.

In this special volume, Postigo and Martínez-Sellés showed that women with HF are more likely to be older, hypertensive, present valvular heart disease, and non-ischemic cardiomyopathy than men. They depicted relevant sex-related differences, including biological mechanisms for HF, age, etiology, precipitating factors, comorbidities, left ventricular ejection fraction, treatment effects, and prognosis. Women have greater clinical severity of HF, with more symptoms and worse functional class, and receive less guideline-proven therapies than men. In spite of both facts, females with HF have better prognosis than males. The authors showed how the reasons for this survival advantage are probably multifactorial but prior pregnancies seem to play a role. López-Vilella et al. describe how female sex confers different prognosis in patients with HF. In 1,291 patients discharged after HF exacerbation, the authors found a trend to better survival in females with reduced ejection fraction than in males. Yet, women presented more readmissions than men, in accordance with the previously described greater clinical severity in females.

Cediel et al. described sex-related differences in HF biomarkers. Kinetics of biological circulating biomarkers are different in women and men but most clinicians do not take sex into account when they assess them. Women tend to exhibit higher levels of natriuretic peptides and galectin-3 and lower levels of cardiac troponins and soluble ST2 than man. Many biological factors explain these differences, including body composition, fat distribution, or menopausal status.

Farrero et al. focused on the impact of HF therapies in women. Current HF guidelines recommend drug up-titration to the same target doses in both men and women, but some factors may impair achieving this goal in women, among them, more common adverse drug reactions and lack of evidence regarding the optimal drug dose. Women are less likely than men to receive a cardiac device in clinical practice, although they show better response to cardiac resynchronization therapy. Females also receive advanced HF therapies less frequently. Technological advances in mechanical circulatory support, with smaller devices, will likely increase their implantation in women. Tokodi et al., used a machine learning approach in a registry of 2,191 patients treated with cardiac resynchronization therapy. The authors were able to create models that predicted all-cause mortality. Interestingly, sex-specific patterns of predictors were identified, and hemoglobin was less important in females compared to males. Chibber and Baranchuk revised sex-related differences in catheter ablation for HF patients with atrial fibrillation. These differences include the referral of fewer women for catheter ablation, older age of women at ablation, and higher risk of post-ablation recurrence of atrial fibrillation.

García-Cosío et al. focused on sex influence in advanced HF therapies and outcome following heart transplantation. The authors described how women account for a minority of patients on the waiting list for heart transplantation or other advanced HF therapies. However, long-term results of heart transplants are equal for both men and women. Ayesta described the influence of sex-mismatch on prognosis after heart transplantation. In most studies, donor/recipient sex-mismatch has been associated with poor prognosis, especially in male recipients of female hearts. This is probably related to physiological sex-related differences, differences in complications rates after heart transplantation (rejection, cardiovascular allograft vasculopathy, and primary graft failure), size mismatch, and recipient pulmonary hypertension. The author concluded that, when allocating a graft, sex-mismatch should be considered.

Finally, Sobanski et al. described sex-related differences in palliative care for HF patients. Women live longer, and after a husband or partner's death, they suffer from a stronger sense of loneliness, are more dependent on institutionalized care and have more unaddressed needs than men. As the prevalence of comorbidities [like diabetes (4) or chronic pain syndromes] grows with age, women suffer from a higher number of symptoms (such as pain and breathlessness) than men. Sex-specific differences have been described in symptom pathophysiology, distribution and the required management needed for their successful alleviation.

This Research Topic highlights the importance of studying sex differences in HF and provide insight on factors that may contribute to future studies regarding the role of sex in cardiovascular physiology and HF pathophysiology. Further data may help to improve the diagnosis and management of HF in women and men.

## Author Contributions

All authors listed have made a substantial, direct and intellectual contribution to the work, and approved it for publication.

## Conflict of Interest

The authors declare that the research was conducted in the absence of any commercial or financial relationships that could be construed as a potential conflict of interest.
